# Risk-benefit analysis of surgical treatment strategies for cystic craniopharyngioma in children and adolescents

**DOI:** 10.3389/fonc.2024.1274705

**Published:** 2024-01-16

**Authors:** Michael Schmutzer-Sondergeld, Stefanie Quach, Sebastian Niedermeyer, Nico Teske, Moritz Ueberschaer, Christian Schichor, Mathias Kunz, Niklas Thon

**Affiliations:** Department of Neurosurgery, LMU University Hospital, LMU Munich, Munich, Germany

**Keywords:** craniopharyngioma, stereotaxy, cyst drainage, microsurgery, endocrinological outcome, visual outcome

## Abstract

**Objective:**

Treatment strategies for craniopharyngiomas are still under debate particularly for the young population. We here present tumor control and functional outcome data after surgical treatment focusing on stereotactic and microsurgical procedures for cystic craniopharyngiomas in children and adolescents.

**Methods:**

From our prospective institutional database, we identified all consecutive patients less than 18 years of age who were surgically treated for newly-diagnosed cystic craniopharyngioma between, 2000 and, 2022. Treatment decisions in favor of stereotactic treatment (STX) or microsurgery were made interdisciplinary. STX included aspiration and/or implantation of an internal shunt catheter for permanent cyst drainage. Microsurgery aimed for safe maximal tumor resections. Study endpoints were time to tumor recurrence (TTR) and functional outcome including ophthalmological/perimetric, endocrinological, and body-mass index (BMI) data.

**Results:**

29 patients (median age 9.9 yrs, range 4-18 years) were analyzed. According to our interdisciplinary tumor board recommendation, 9 patients underwent stereotactic treatment, 10 patients microsurgical resection, and 10 patients the combination of both. Significant volume reduction was particularly achieved in the stereotactic (p=0.0019) and combined subgroups (p<0.001). Improvement of preoperative visual deficits was always achieved independent of the applied treatment modality. Microsurgery and the combinational treatment were associated with higher rates of postoperative endocrinological dysfunction (p<0.0001) including hypothalamic obesity (median BMI increase from 17.9kg/m^2^ to 24.1kg/m^2^, p=0.019). Median follow-up for all patients was 93.9 months (range 3.2-321.5 months). Recurrent tumors were seen in 48.3% and particularly concerned patients after initial combination of surgery and STX (p=0.004). In here, TTR was 35.1 ± 46.9 months. Additional radiation therapy was found indicated in 4 patients to achieve long-lasting tumor control.

**Conclusion:**

In children and adolescents suffering from predominantly cystic craniopharyngiomas, stereotactic and microsurgical procedures can improve clinical symptoms at low procedural risk. Microsurgery, however, bears a higher risk of postoperative endocrine dysfunction. A risk-adapted surgical treatment concept may have to be applied repeatedly in order to achieve long-term tumor control even without additional irradiation.

## Introduction

Craniopharyngiomas are benign, slowly growing tumors of the midline with an incidence of 0.2-0.5/1.000.000 (1–5). 30-50% of tumors affect children and adolescents. Patients may present with gradual onset (impaired vision, endocrinologic deficiencies) or (sub-) acute symptoms due to impaired cerebrospinal fluid circulation. Initial diagnosis is based on magnetic resonance imaging (MRI), which confirms either solid, mixed, or predominantly cystic tumor formations in the sellar/perichiasmatic region. Complex involvement of the optic system, vascular structures, hypothalamus, and pituitary stalk often must be critically evaluated for both symptom burden and treatment considerations. Treatment aims for functional preservation and long-lasting tumor control. Therapy gives priority to surgical procedures over radiation therapy (6–9). The value of drug-based tumor therapy is controversial (10–13).

In case of space-occupying cyst formations, stereotactic procedures may be a minimally invasive alternative to microsurgical tumor resections (14, 15). Compared to sole aspiration, the stereotactic implantation of an internal shunt catheter allows the permanent drainage of the cysts into the ventricular system and basal cisterns. These minimal-invasive procedures are associated with low intra- and perioperative risks, however recurrent cystic formations may occur. Endocrinological functions after stereotactic surgery have been shown to be superior to conventional microsurgery (16). For the pediatric and adolescent population, efficacy and risk profile of minimally invasive stereotactic procedures for cystic craniopharyngiomas have not been explored in the context of the therapeutical armamentarium yet. Especially outcome parameters like hypothalamic deficiency, changes of body mass index (BMI) or psychosocial consequences like graduation and occupation are of special interest in this patient group. Thus, we performed a retrospective study of pediatric and adolescent patients suffering from predominantly cystic craniopharyngiomas analyzing both stereotactical and microsurgical treatment concepts.

## Methods

### Patient population

After approval of the institutional review board of the Ludwig-Maximilians-University in Munich (reference number 19-798), the tumor registry of the Department of Neurosurgery was searched for all consecutive patients less than 18 years of age undergoing any surgical treatment of newly-diagnosed cystic craniopharyngiomas between, 2000 and, 2022. Clinical and diagnostic evaluations were collected preoperatively and within 7 days after surgery as well as at routine follow-up evaluations (normally 6 weeks, 12 months, 24 months and later). Functional outcome analyses referred to pre-operatively obtained data. All patients respectively their parents gave informed consent before surgical treatment.

### Magnetic resonance imaging

According to our standard in-house protocol, the magnetic resonance imaging (MRI) (1.5- or 3.0-T scanners: Magnetom Symphony, Siemens, Erlangen; Signa HDxt; GE Healthcare, Little Chalfont, United Kingdom) routinely included axial T2-weighted sequence (with slice thickness of 2 mm), 3-dimensional T1-weighted sequences before and after intravenous administration of gadopentetate dimeglumine (0.1 mmol/kg body weight; Magnevist; Schering Corporation, Kenilworth, NJ), and constructive interference in steady-state sequences (CISS, with slice thickness of 1 mm), with axial, sagittal and coronal reconstructions each. Volumetric tumor analyses of pre- and post-operative MR images were performed by semi-manual segmentation of pre- and post-operative T2/CISS and contrast-enhanced (CE) T1 images and using a commercially available software tool (SmartBrush^®^, Elements^®^, BRAINLAB AG, Munich, Germany). For study inclusion, only craniopharyngiomas with at least 70% cystic tumor volume were included. This results in a relevant solid tumor fraction of about 10-30% of the tumor volume.

### Ophthalmological protocol

Routine ophthalmological examinations (including visual acuity measurements and perimetry of the visual field) were obtained by ophthalmologists pre-operatively and at routine follow-up intervals. Deficits in visual acuity were classified as mild (0.9-0.5) or severe (0.4-0), visual field deficits as partial anopsia or complete hemianopsia.

### Endocrinological protocol, metabolic, and psychosocial outcome

Pre-operatively and at defined time points (6 weeks, 12 months, 24 months and later) after surgery, basal serum levels of growth hormone, insulin-like growth factor 1, adrenocorticotropic hormone, baseline cortisol, prolactin, luteinizing hormone, follicle-stimulating hormone, thyrotropin and thyroid hormone (fT3 and fT4), testosterone and estradiol were measured and evaluated by an endocrinologist. Neurohypophyseal function was measured by levels of serum sodium and osmolarity, fluid intake and output and urine specific gravity level. Endocrinological deficits were pre- and post-operatively classified as anterior or posterior hypopituitarism, which includes complete and incomplete insufficiencies, accordingly to the impacted lobe. In case of partial or complete involvement of both lobes, insufficiency was defined as panhypopituitarism. Pre- and post-operatively (at last FU time point) patients´ BMI was calculated with the following formula: bodyweight (in kg)/(body height (in m))^2^. To determine psychosocial outcome parameters, graduation level, occupation and the existence of neurocognitive deficits were documented by follow-up telephone interview.

### Treatment protocol

According to our in-house protocols surgical treatments included microscopically/endoscopically assisted transsphenoidal microsurgical resection for sellar, subchiasmatic tumors. Suprachiasmatic pure cystic tumors underwent stereotactic treatment. Suprachiasmatic, mixed tumors exhibiting both cystic and solid compounds were treated either by microsurgery via a subfrontal and/or pterional approach or stereotactic technique depending on tumor configuration and risk assessment of the surgeon.

**Figure 1:**
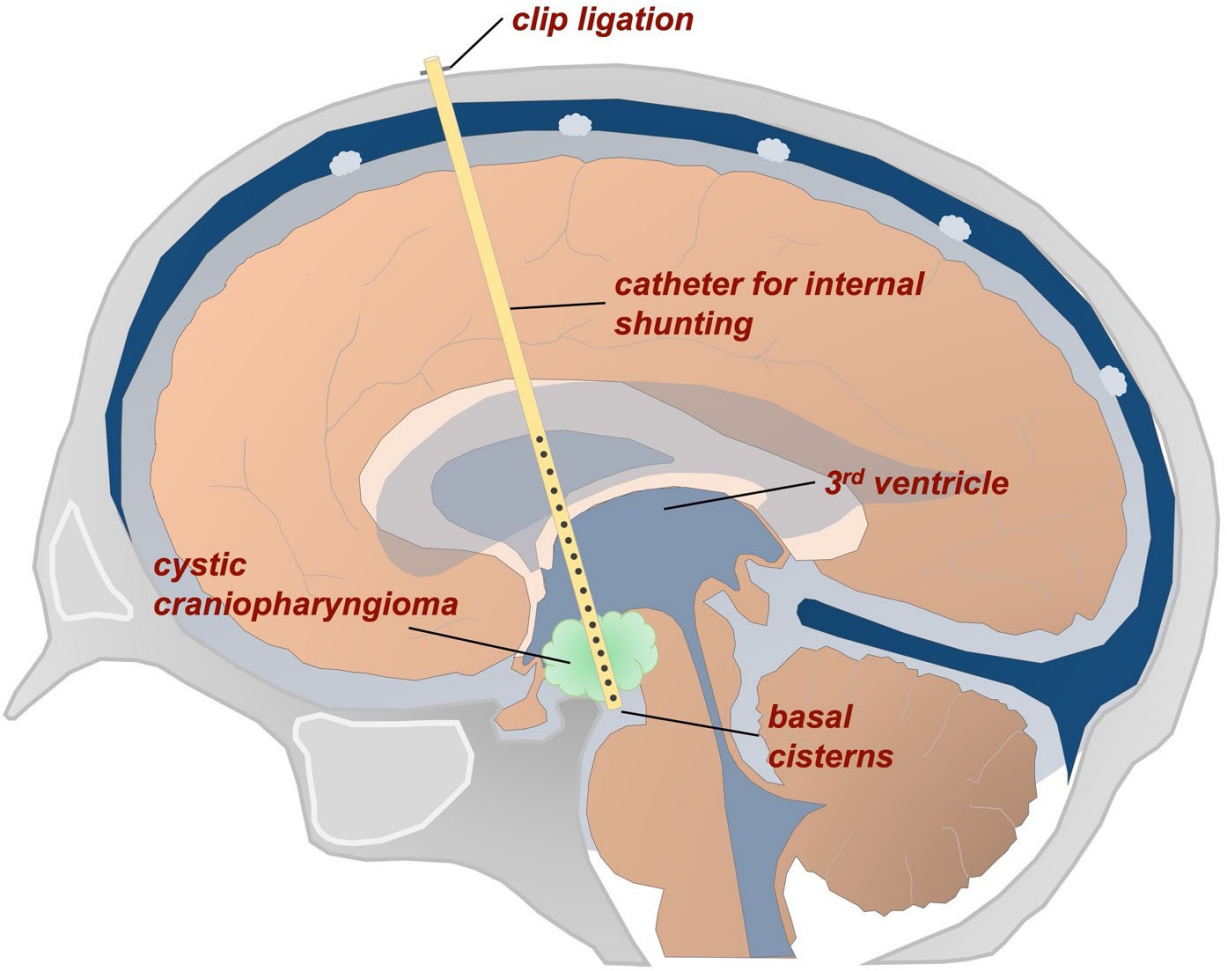
Schematic representation of a stereotactic internal shunt catheter implanted to connect the cystic tumor with the ventricles and basal cisterns.

For stereotactic treatment, an internal shunt catheter was implanted to connect the cystic tumor to both the supratentorial ventricular system (upstream drainage) and the basal cisterns (downstream drainage) (**Figure 1**).

Treatment planning (iPlan stereotaxy; Brainlab, Munich, Germany) was based on a stereotactically localized contrast-enhanced computed tomography (CT) scan (0.6 mm slice thickness) and the pre-operative MRI data (T1-weighted without contrast, T2-weighted/CISS sequences, contrast-enhanced magnetic resonance angiography), which were co-localized with the CT scan. The Leksell^®^ Coordinate Frame G (Elekta GmbH Hamburg, Germany) was applied for stereotactic treatment. A 1.3 mm diameter catheter (Becker EDMS ventricular catheter, Barium Impregnated; Medtronic Inc, Minneapolis, USA) was stereotactically implanted via a 2 mm burr hole. Additional catheter perforations were added manually to allow optimal up- and downstream drainage. This technique has been specified and published in our and also other neurosurgical departments through years of experience (16–20). The catheter was fixed extracranially with a hemoclip (Titanium Ligation-Clip, 150mm length, B Braun, Melsungen, Germany) placed orthogonally on the catheter on the calvaria preventing the catheter from sliding into the brain. Above this a TachoSil® (Takeda Pharmaceuticals, Konstanz, Germany) was attached for adequate closure and additional fixation. Before catheter implantation, the cystic fluid was washed out by successive aspiration and instillation of sterile isotone sodium chloride solution. The stereotactic implantation of a permanent catheter is aimed at permanent drainage of a tumor cyst; removal is not usually intended. In fact, catheter systems can become adherent after years due to scar strands and can only be removed with a significantly increased risk. No catheter was removed in this series either.

### Outcome analyses

Surgical results and follow-up analyses were assessed by quantitative MRI volume measurements. In case of complete resection/drainage, any detectable solid/cystic tumor recurrence was classified as tumor progression. Otherwise, an increase of the postoperative tumor/cystic volume of more than 25% or any development of associated clinical symptoms was defined as progression. A post-operative increase in visual acuity of >0.2 and/or a decreased visual field defect was defined as an improvement, whereas a post-operative decrease in visual acuity of >0.2 and/or an increased visual field defect was assumed as deterioration. Otherwise, visual performance was classified as unchanged. With respect to endocrine function, post-operative improvement was assumed in case of complete recovery of at least one affected hormonal axis plus a stable status of the remaining axes. A new complete/partial insufficiency of each hormonal axis post-operatively was classified as deterioration. Otherwise the endocrinological function was classified as unchanged.

### Risk assessment

Perioperative morbidity rates were determined according to all documented medical, neurological, and approach-related adverse events. Transient and permanent deficits were differentiated. Functional morbidity was analyzed separately.

### Statistical methods

The reference point of this study was the date of first surgery. Last follow-up date was October, 2022. Primary endpoint was date of tumor progression, secondary endpoints included functional outcome and treatment-related adverse events. Time to tumor recurrence (TTR) was analyzed by using the Kaplan-Meier method. To compare the survival curves, the log-rank test was used. Results were tested by using a 2-way analysis of variance- (ANOVA), Student´s t- and Fisher´s exact test.

Risk factor analysis for a functionally worsened outcome was conducted by a univariate test. Deterioration in any category was defined as worsened functional outcome.

GraphPad PRISM8.0d software was used for statistical analysis (GraphPad, San Diego, CA, USA). Statistical significance was set at p<0.05.

## Results

Twenty-nine patients were included (11 females; median age 9.9 years, range 1.8-18 years). Leading clinical symptoms at first diagnosis were headache in 25 (86.2%), diplopia in 19 (65.6%), visual impairment in 10 (34.5%) and/or visual field deficiencies in 11 (37.9%), and endocrinological dysfunction in 8 (27.6%) patients, respectively (see patients characteristics in **Table 1**). In 24 patients (82.7%) BMI was documented and revealed a median value of 22.3kg/m^2^ (range, 13.7-27.8 kg/m^2^).

**Table 1:** Patient characteristics and distribution of symptoms at initial diagnosis.

Parameters	Stereotaxy	Microsurgery		Combination	p-value
Patient characteristics					
**Total, n (%)**	9 (31.0)	10 (34.5)		10 (34.5)	
**Sex, n (%)**					
**Female**	3 (10.3)	6 (20.7)		2 (7.0)	0.2
**Male**	6 (20.7)	4 (13.8)		8 (27.6)	0.4
**Age (yrs)**					
**Mean ± SD**	8.9 ± 4.2	11.2 ± 4.3		8.8 ± 4.0	0.39
**BMI (kg/m^2^)**	23.9	18.8		19.0	0.05
**Tumor volume**	83.2 ± 47.4cm^3^	38.6 ± 31.3cm^3^		96.3 ± 46.3cm^3^	**0.01**
**Displacement of chiasm, n (%)**	6 (20.7)	8 (27.6)		8 (27.6)	0.7
Symptoms					
**Headache, n (%)**	9 (31.0)	8 (27.6)		8 (27.6)	0.3
**Diplopia, n (%)**	5 (17.2)	5 (17.2)		9 (31.0)	0.1
Visual acuity, n (%)					
**No deficit**	6 (20.7)	7 (24.1)		6 (20.7)	0.9
**Mild deficit**	3 (10.3)	3 (10.3)		1 (3.4)	0.4
**Severe deficit**	0	0		3 (10.3)	**0.04**
Perimetry, n (%)					
**No deficit**	5 (17.2)	7 (24.1)		6 (20.7)	0.8
**Partial anopsia**	2 (7.0)	1 (3.4)		1 (3.4)	0.7
**Hemianopsia**	2 (7.0)	2 (7.0)		3 (10.3)	0.9
Endocrinology, n (%)					
**No deficit**	9 (31.0)	6 (20.7)		6 (20.7)	0.08
**Anterior hypopituitarism**	0	2 (7.0)		4 (13.8)	0.09
**Posterior hypopituitarism**	0	2 (7.0)		0	0.1
**Panhypopituitarism**	0	0		0	0.99

Significant values are shown in bold.

Nine (31.0%) tumors were predominantly located within the sellar region, 20 (69.0%) tumors exhibited a suprasellar, intradural tumor component with a relevant solid tumor component in 3 (10.3%) patients. Significant compression of the chiasm was seen in 22 (75.9%) tumors. All tumors were of adamantinomatous subtype. Overall, median tumor volume was 73.2 cm³ (range 5.1-145.7cm^3^). Initial tumor volume was smaller in the microsurgical group (38.6 ± 31.3cm^3^) compared to the stereotactic group (83.2 ± 47.4cm^3^) as well as the combined treatment group (96.3 ± 46.3cm^3^) (p=0.01).

### Surgical treatment

Nine patients underwent stereotactic treatment alone (3 patients for initial cyst aspiration and 6 patients for catheter implantation). In 10 patients the tumors were resected, and 10 patients were scheduled for the combination of both treatment modalities. These three surgical subgroups did not differ with respect to age and clinical symptom burden (see **Table 1**).

**Figure 2:**
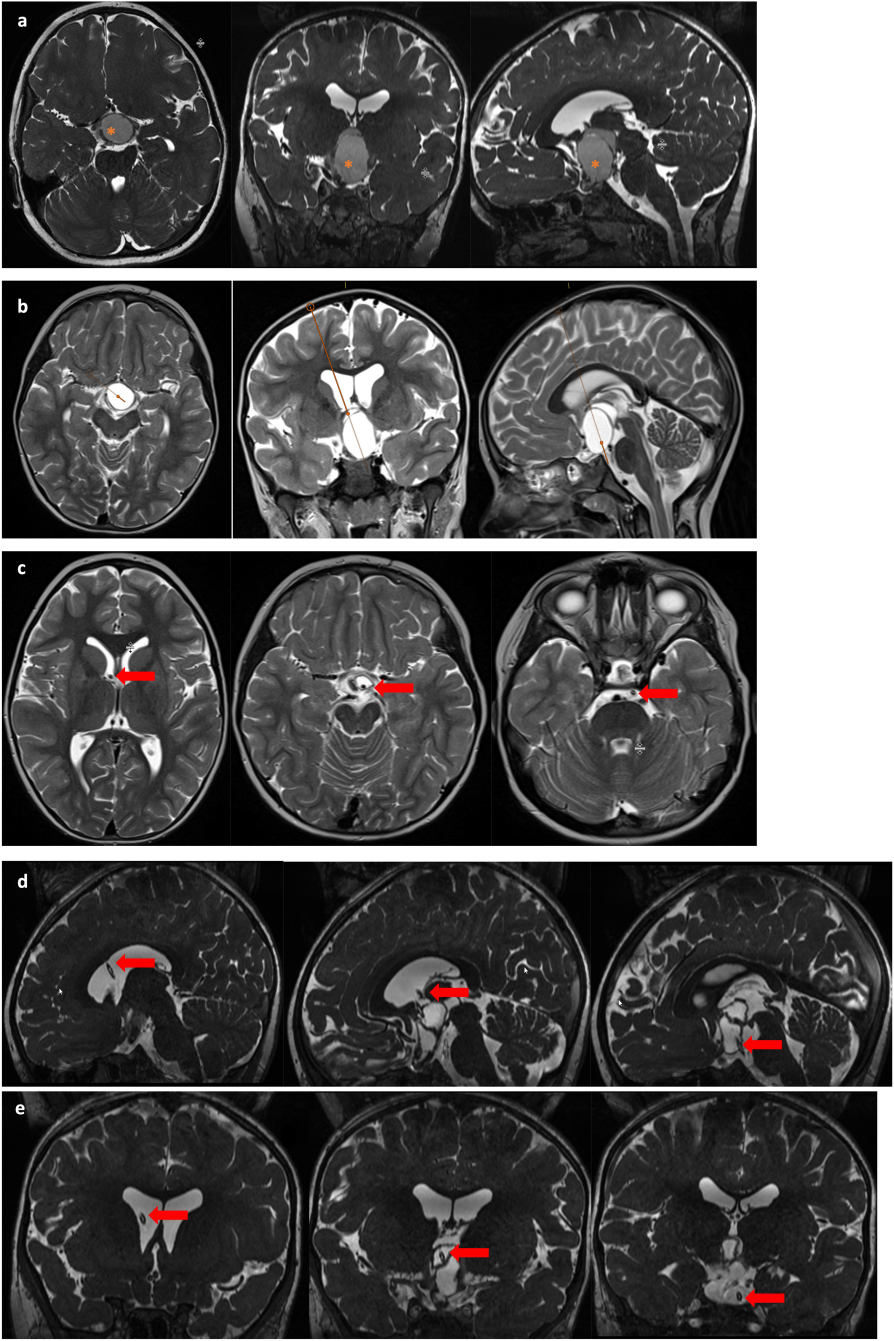
MR imaging of a 5-year old female patient with a suprasellar cystic, space-occupying craniopharyngioma treated with stereotactic implantation of an internal shunt system. This patient presented with initial symptoms of mild visual deficiency and headache. Image line **(A)** demonstrates preoperative findings with a large intra-/suprasellar cystic tumor (*) compressing the chiasma. After planning the stereotactic trajectory (image line **B**) for implantation of an internal shunt system, the cystic tumor formation was reduced (image lines **C** (axial), **D** (sagittal) and coronal **E**) resulting in a decompressed chiasm and consecutive improvement of visual function. The arrow marks the course of the internal shunt through the foramen Monroi, the tumor cyst with final position in the prepontine cistern.

Overall, treatment let to a significant reduction of the median volume to 17.3cm^3^ (range 0.8-96.1cm^3^) (p<0.001). The respective median tumor volumes dropped from 83.2cm^3^ (range 20.6-140.0cm^3^) to 15.2cm^3^ (range 0.86-59.5cm^3^) after catheter treatment (p=0.0019), from 64.2cm^3^ (range 23.6-114.3cm^3^) to 14.7cm^3^ (range 3.5-36.8cm^3^) after cyst aspiration (p=0.2), from 38.7cm³ (range 5.1-90.3cm³) to 12.9cm³ (range 0.9-44.3 cm³) after surgical resection (p=0.65), and from 96.3cm^3^ (range 9.8-145.7cm^3^) to 20.5cm^3^ (range 1.6-96.1cm^3^) after the combined treatment approach (p<0.001). As an example, stereotactic implantation of an internal shunt is shown in **Figure 2**.

Five of the 7 (71.4%) patients with visual disturbance showed postoperative improvement, a new visual deficit was seen in 1 patient (3.4%) (**Table 2**). 18 patients developed endocrine dysfunction after surgery, which was particularly seen after microsurgery (alone or in combination with STX) (p<0.0001) (**Table 2**). Also, a significant increase of the median BMI from 17.9kg/m^2^ to 24.1kg/m^2^ was selectively observed in patients undergoing primary resection and combined treatment (p=0.019).

**Table 2:** Functional outcome according to surgical procedures.

Functional outcome				
	Stereotaxy (n=9)	Microsurgery (n=10)	Combination (n=10)	*p-value*
**Visual Outcome,** **n (%)** ***p-value***	Stable: 7 Worse: 0 Improved: 2	Stable: 8 Worse: 0 Improved: 2	Stable: 8 Worse: 1 Improved: 1	0.99 0.4 0.7
	**0.0015**	**0.0004**	**0.0006**	
**Perimetrical Outcome,** **n (%)** ***p-value***	Stable: 6 Worse: 0 Improved: 3	Stable: 7 Worse: 0 Improved: 3	Stable: 8 Worse: 0 Improved: 2	0.8 0.99 0.8
	**0.01**	**0.004**	**0.0004**	
**Endocrine Outcome,** **n (%)** ***p-value***	Stable: 6 Worse: 3 Improved: 0	Stable: 1 Worse: 7 Improved: 2	Stable: 2 Worse: 8 Improved: 0	**0.02** 0.09 0.1
	**0.01**	**0.0096**	**0.0004**	

Significant values are shown in bold.

### Adjuvant treatment and outcome

**Figure 3:**
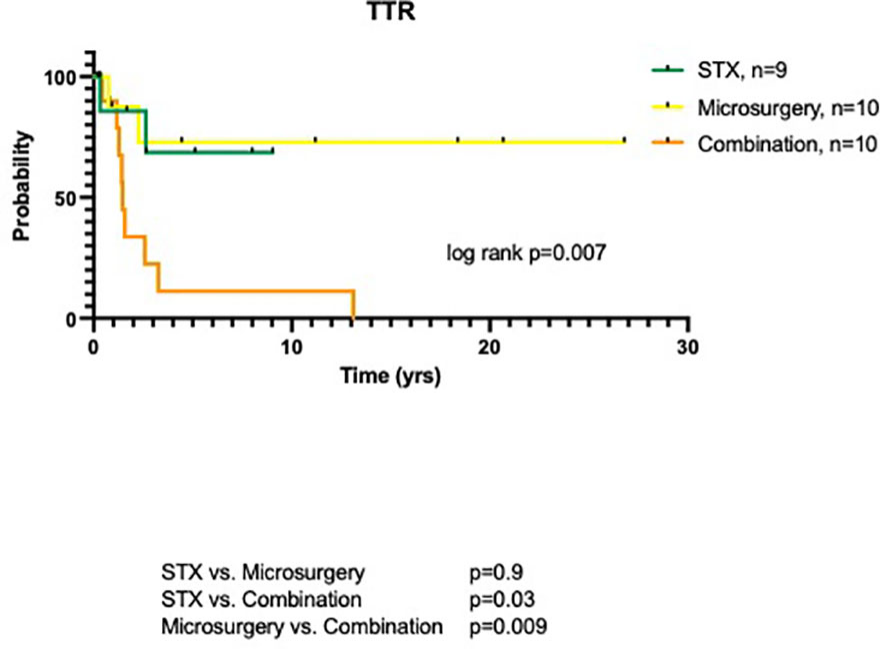
TTR of pediatric craniopharyngioma patients depending on surgical procedure: stereotaxy, microsurgery and combined treatment.

Median follow-up (FU) for all patients was 65.5 months (range 8.0-321.5 months). Mean FU for patients with initial microsurgery (alone or in combination), however, tended to be longer compared to the stereotactic group (123.7 ± 94.6 vs. 41.8 ± 34.6 months; p=0.05). Three patients (2 STX, 1 microsurgery) were lost to FU with periods of less than 3.2 months. Overall, local recurrence was noted in 13 patients (44.8%). This included 2/9 (22.2%) patients after internal shunting, 2/10 (20.0%) patients after microsurgical resection, and 9/10 (90.0%) patients after the combined treatment approach (p=0.002). Overall, time to recurrence (TTR) was 30.0 ± 39.7 months. TTR was 4.0 and 31.8 months in the two cases with tumor recurrence after stereotatic treatment and 8.9 and 27.1 months after microsurgery, respectively. The 9 patients (90%) of the combined surgical group had an average TTR of 35.1 ± 46.9 months (median 17.4 months, range 5.1-157.3 months), with an 1-year probability without tumor recurrence of 11.2% (log rank, p=0.007, **Figure 3**).

### Treatment for recurrent tumors

Recurrent tumors preferentially underwent – if necessary even multiple – additional surgical treatments. This included additional stereotactic catheters in 2 recurrent cystic tumors using a different trajectory, both belonging to the initial stereotactic group. Microsurgical re-resections were done in 2 patients, all having been operated before. Four patients (13.8%) underwent additional external beam radiation after a median period of 79.2 months (range 17-216 months). These patients had a median age of 5.2 years (range 4-7 years) at first diagnosis and belonged to the combined treatment (3 patients, each 2 microsurgical and 1 stereotactic procedures before) and the stereotactic groups (1 patient, 4 stereotactic procedures before), respectively. Initial tumor volumes were greater compared to tumors without the need for additional external beam radiation (145.7cm^3^ vs. 57.6cm^3^; p=0.015) and each tumor had a significant solid component. Patients undergoing adjuvant external beam radiation showed a significant increase of the BMI (22.3kg/m^2^ to 30.9kg/m^2^; p=0.04) during the course of disease. With regard to the remaining endocrinological function, 2 patients showed a complete pituitary insufficiency after irradiation, whereas the other 2 patients had no disturbance (p=0.3)

### Surgical morbidity

In an overall of 56 surgical interventions, four patients (7.1%) developed a transient perioperative morbidity. This included one CSF leak (1.8%) and one wound healing disorder (1.8%) after stereotactic treatment as well one indication for ventricular-peritoneal shunt implantation (1.8%) after microsurgery and one patient with multilocular ischemia (1.8%) after opening of the ventricles and cyst resection without statistical significance between treatment groups (p=0.4). No mortality was observed.

### Risk analysis

In univariate analysis, larger pre- and postoperative cyst volumes and higher numbers of interventions were associated with worsened functional outcome. The choice of surgical approach (STX vs. microsurgery vs. combined treatment) did not affect functional outcome significantly (for details see **Table 3**).

**Table 3:** Univariate analysis for functional outcome.

Characteristic	Odds ratio	Univariate 95% CI	p-value
Functionally worsened outcome			
Cyst volume pre-OP	7.994	-0.7420 to -0.03107	**0.04**
Cyst volume post-OP	11.352	-0.8302 to -0.2589	**0.003**
Number of interventions	8.434	-0.7215 to -0.01144	**0.04**
Surgical procedure	1.355	-0.3235 to 0.4937	0.6
(STX vs. Microsurgery vs. combination)			
BMI pre-OP	1.179	-0.4586 to 0.4750	0.9
BMI post-OP	1.963	-0.6329 to 0.2598	0.3
Age at first surgery	1.225	-0.4441 to 0.3793	0.8
Sex (m vs. f)	1.096	-0.4408 to 0.3828	0.9

Significant values are shown in bold.

### Socioeconomic outcome

For 23 (79.3%) of the included patients socioeconomic outcome parameters could be determined at long-term FU evaluations (5 lost to follow-up). Two patients (8.7%) had slight educational impairments, but were enrolled in regular school settings. These patients belonged to the combined treatment group. Twenty-one patients (91.3%) reported no insufficiencies postoperatively and were attending appropriate institutions to obtain the highest educational qualification.

## Discussion

Craniopharyngioma is a rare skull base tumor that originates in the sellar region and occurs predominantly in children and adolescents (2–5, 21). Proximity to eloquent neurovascular and endocrine structures may cause visual deficits and hormonal disturbances. The latter may lead to long-term growth retardation (22–24). While these symptoms are often overlooked, particularly in young patients, impaired CSF circulation can lead to more rapid deterioration including headache, nausea, epileptic seizures, and decreased level of consciousness (11, 25). Acute symptoms are typically seen in more cystic craniopharyngiomas, as their volume may change more rapidly (14, 15, 26).

The cohort of patients with cystic craniopharyngioma presented in here matches the literature with respect to age distribution and clinical symptoms (22, 27–29). Frequency of preoperative anterior or posterior pituitary deficits, however, was slightly lower, which particularly concerned the subgroup with stereotactic drainage (16, 22). Preoperative cyst volumes were significantly greater in both stereotactic groups (alone or with additional resection), while significant volume reduction was achieved in all these cases (p<0.001 each). The aim of minimally invasive stereotactic catheter implantation was to reduce the volume of the craniopharyngioma cyst by creating a stoma with permanent drainage of the cyst contents into the ventricular system and the basal cisterns. This stoma does not require repeated irrigation, as would be technically possible with an Ommaya reservoir, for example. Furthermore, in our hands there was no indication for local application of a drug via a corresponding reservoir. For this reason, we did not use a subcutaneously applied Ommaya reservoir, but we do see the possibility of switching to this modification if indicated (10, 30). The sometimes thick and tough cyst wall in cystic craniopharyngiomas can usually be perforated during stereotactic catheter implantation through the probe advanced to the target point so that the inner shunt catheter is placed through the holes in the craniopharyngioma cyst. In the open resected craniopharyngioma patients, we only performed a pure tumor cyst resection and did not implant a catheter. However, this option is an important additional aspect that should be considered for further open resections, especially if the tumor cysts can only be removed incompletely.

In contrast, volume reduction after microsurgery alone was less effective (p=0.65) and differed from previous reports (6, 16). There is probably a bias due to the alternative surgical strategies in our monocentric cohort, as the microsurgical group particularly contained smaller cyst formations. It appears that these tumors were more frequently located intrasellar. This localization is considered to be more suitable for transsphenoidal resection and less for stereotactic catheter procedures. Nevertheless, clinical improvement, particularly of visual disturbances, was also achieved in this surgical subgroup.

To date, microsurgical resection has been considered the gold standard in the treatment of craniopharyngiomas. Several studies, however, critically discuss the benefits and disadvantages particularly for cystic tumors and craniopharyngiomas in children (31–36). Due to the interdigitation with the hypothalamus, postoperative endocrinological disorders are of great concern (37–39) mainly after radical microsurgical resections. This affects especially the pediatric subpopulation with profound endocrinological insufficiency, hypothalamic obesity, neurocognitive deficits, behavioral disorders, and reduced quality of life (33, 40–43). More gentle surgical treatment options, however, are prevalently associated with significant recurrence rates and the need for adjuvant radiation (40, 44). In our study cohort, a differential surgical approach was well tolerated, only a few procedure-related complications were seen, all being minor and transient in nature. Cumulative morbidity after stereotaxy (3.6%) and microsurgery (3.6%) proved to be lower than previously described (16, 45). Furthermore, after microsurgical craniopharyngioma resection with ventricular opening, multiple infarcts were found in the postoperative course, especially in the frontal, temporal and also occipital lobes, assuming that this irritation with a ventriculitis-like course probably developed due to the cyst fluid entering the ventricular system, which led to a kind of vasospasm. Functional outcome analyses revealed stable postoperative results in all 3 groups in terms of visual and perimetric outcome. Endocrinological dysfunction, however, tended to be more pronounced in the microsurgical subgroup. This is in line with previous reports on cystic craniopharyngiomas in adults showing that the transcranial microsurgical approach leads to distinctive endocrinological deterioration compared to stereotactic treatment (16, 46). This also included worsened long-term BMI measurements after microsurgery.

In the literature gross total resection has been associated with tumor control rates of 65-90% compared to subtotal resection with inferior rates of 10-50% (6, 25, 47, 48). In our cohort long-term tumor control was achieved in 15/29 patients (51.7%). Fourteen patients, however, developed a recurrence within the FU period. Accordingly, tumor control seems to differ between predominantly solid/partially cystic and predominantly cystic craniopharyngiomas. Today, the reason for these differences in growing behavior are not fully understood, however, they may be the result of different subgroups of tumors with different molecular profiles and biological behavior (31, 49).

Notably, the intrinsic growth behavior of tumors was maintained at recurrence, so that mainly cystic tumors develop new cysts, while partially solid tumors preferentially showed solid tumor growth. As a consequence, in the vast majority of cases the initial surgical strategy was maintained also at recurrence (16, 50). Tumors recurred in two patients each after initial microsurgery or stereotaxy, always within 20 months after first surgery. Similar observations were seen in the cohort presented by Rachinger and coworkers focusing on stereotactic versus microsurgical procedures in adults (16, 29). However, we clearly have to mention that endoscopic approaches and procedures increasingly have replaced the microscopic therapy focusing on broader possibilities for surgical approaches (51). In here, there was also no significant difference in a direct comparison of these two therapeutic procedures even at significantly longer median TTRs (16). Most tumor recurrencies, however, were seen in the subgroup of patients who were selected for an *a priori* combined surgical management. In here, 9/10 patients developed recurrent cyst and/or solid tumor formations.

One of the main goals in the therapy of especially recurrent craniopharyngioma is to avoid or at least withhold percutaneous adjuvant radiotherapy in the young population. Due to the proximity to eloquent brain areas and the interdigitation with the hypothalamus, preserving functionality and proper neurological and endocrinological development is a crucial aspect for therapy planning (11–13, 25, 44, 47). Here, a remarkable low rate of only 4 patients (13.8%) underwent percutaneous radiotherapy after multiple therapies in advance. These patients had a larger median tumor volume than those without the need for adjuvant radiation (145.7cm^3^ vs. 57.6cm^3^; p=0.015). Furthermore, it is imperative to mention that due to the only small cohort of patients in our study and of these only 4 patients who underwent adjuvant radiation, a truly tangible negative effect of radiation cannot and should not be concluded as radiation therapy reflects an important key in the multidisciplinay treatment of pediatric craniopharyngioma (44, 52, 53). Rather, our study was intended to demonstrate the different stereotactic and surgical options for cystic infantile craniopharyngiomas so that the effect and endocrionological outcome must be considered in the context of a disease necessitating multidisciplinary therapy.

### Limitations

Due to the retrospective design of this study with a comparable short FU period, only limited conclusions concerning the place of the three surgical strategies as well their risk profiles and long-term morbidity can be drawn from these data, as an effect of selection bias cannot be excluded. Moreover, functional outcome parameters could only be drawn from pre- and postoperative time points at last follow-up. Further psychosocial and intellectual developmental parameters could not be recorded in a prospective purpose so that exact statements of neurocognitive and behavioral aspects after surgical treatment of childhood/adolescent craniopharyngiomas are impossible. Due to the small case number, a multivariate analysis did not appear useful in our study cohort, so only a univariate analysis could be performed. In this respect, further prospective and even multicenter studies may allow multivariate risk assessment.

## Conclusions

This is, to our knowledge, one of the largest studies to compare various surgical procedures for mainly cystic/predominant cystic craniopharyngiomas in a pediatric/adolescent population. Both stereotactic and microsurgical procedures turned out to be safe and effective to achieve volume reduction and clinical improvement. However, the risk of postoperative hypothalamic/endocrine dysfunction was lower in case of stereotactic intervention. The respective tumor control rate did not differ from transcranial resection. The actual reason for this positive effect of a stereotactic procedure remains unclear so far. It can be suggested that the slow upstream- and downstream-drainage of the tumorcyst into the ventricle and prepontine cistern may play an essential role, compared to sudden cyst collapse or manipulation of cystic parts adhering to the hypothalamus in locally extended craniopharyngioma in microsurgical resections. In summary, stereotactic cyst drainage enlarges the therapeutic platform for cystic/predominantly cystic craniopharyngioma in a pediatric/adolescent population, however the important key role of radiation therapy in craniopharyngioma should not be disregarded.

## Data availability statement

The raw data supporting the conclusions of this article will be made available by the authors, without undue reservation.

## Author contributions

MS-S: Conceptualization, Data curation, Formal Analysis, Investigation, Methodology, Writing – original draft, Writing – review & editing. SQ: Writing – review & editing. SN: Writing – review & editing. NTe: Writing – review & editing. MU: Writing – review & editing. CS: Writing – review & editing. MK: Data curation, Supervision, Validation, Writing – review & editing. NTh: Data curation, Supervision, Writing – review & editing.
